# Percutaneous anterior C1/2 transarticular screw fixation: salvage of failed percutaneous odontoid screw fixation for odontoid fracture

**DOI:** 10.1186/s13018-017-0640-x

**Published:** 2017-09-29

**Authors:** Ai-Min Wu, Hai-Ming Jin, Zhong-Ke Lin, Yong-Long Chi, Xiang-Yang Wang

**Affiliations:** Department of Spine Surgery, Zhejiang Spine Surgery Center, Orthopaedic Hospital, The Second Affiliated Hospital and Yuying Children’s Hospital of Wenzhou Medical University, 109# XueYuan Western Road, Wenzhou, Zhejiang 325027 China

**Keywords:** Odontoid fracture, Atlantoaxial, Transarticular screw, Minimally invasive, Percutaneous

## Abstract

**Background:**

The objective of this study is to investigate the outcomes and safety of using percutaneous anterior C1/2 transarticular screw fixation as a salvage technique for odontoid fracture if percutaneous odontoid screw fixation fails.

**Methods:**

Fifteen in 108 odontoid fracture patients (planned to be treated by percutaneous anterior odontoid screw fixation) were failed to introduce satisfactory odontoid screw trajectory. To salvage this problem, we chose the percutaneous anterior C1/2 transarticular screw fixation technique in treatment of these patients. The visual analogue score (VAS) of neck pain and Neck Disability Index (NDI) of all patients were scored at pre-operation, 3 months after operation, and final follow-up. Additional, technique-related complications were recorded and collected.

**Results:**

Percutaneous C1/2 transarticular screw fixation was performed successfully in all 15 patients whose odontoid screw fixation failed. No technique-related complications (such as nerve injury, spinal cord injury, and esophageal injury) occurred. The VAS of neck pain and NDI score improved significantly (*P* = 0.000) after operation, and no significant differences were found when compared to 93 non-salvage patients who successfully performed the percutaneous anterior odontoid screw fixation. No screw loose or breakage occurred, all of the odontoid fractures achieve radiographic fusion, bony fusion bridge could be observed at the C1/2 lateral articular facet on 9/15 patients.

**Conclusions:**

We suggest that percutaneous anterior C1/2 transarticular screw fixation is a good alternative salvage technique if percutaneous odontoid screw fixation failed, and it is a minimally invasive, feasible, and safe technique.

## Background

Odontoid fractures are not uncommon injury, accounting for 9–18% of cervical spine fractures [1, 2], especially in elderly patients. Although the conservative methods were well described in treatment of odontoid fractures, type II fractures and shallow type III odontoid fractures [3] (according to the classification of Anderson and D’Alonzo) were recognized mechanically unstable with a high risk of nonunion or mortality [4, 5], and surgical stabilization was recommended [2, 6].

Many posterior surgical techniques were reported in previous literatures [7–9], including Gallie’s C1/2 wiring [10] and Magerl’s posterior C1/2 transarticular screw fixation and posterior C1 lateral mass + C2 pedicle screw fixation [11, 12]; however, these posterior techniques were performed via an open surgical approach, with disadvantages of considerable tissue trauma, blood loss, higher risk of vertebral arteries injury, and some other risks of pneumonia, acute respiratory distress syndrome, and decubitus ulcer [13]. Anterior odontoid screw fixation was first described by Bohler in 1980s [14], proved anatomic feasibility [15], and could preserve the C1/2 rotation and provide adequate stability [16, 17]. Furthermore, anterior odontoid screw fixation could be introduced percutaneously [18–20], with only about 10 mm skin wound, and have the advantages of being minimally invasive, having less blood loss, having shorter skin scar, and having quicker post-operative recovery.

However, anterior odontoid screw fixation is not suitable for all patients. In Grauer type IIC fracture [21], which is extending from anterior-inferior to posterior-superior or with significant comminution, the bone of C2 body for screw anchor is too small to introduce the odontoid screw [22, 23]. Sometimes, the trajectory of odontoid screw cannot be determined satisfactorily [24], such as the case reported by Salem et al. [25] that the fracture position was unacceptable, and other surgical techniques need to be considered [25, 26].

## Materials and methods

### Patient population

Between June 2006 and August 2012, 15 (male, 12 cases; female, 3 cases) in 108 odontoid fracture patients (planned to be treated by percutaneous anterior odontoid screw fixation) were failed to introduce satisfactory odontoid screw trajectory. To salvage this problem, we performed percutaneous anterior C1/2 transarticular screw fixation in treatment of these 15 patients. Eleven cases were type II odontoid fractures and four were shallow type III fractures according to the Anderson and D’Alonzo classification system [3], and three cases were type II B, eight cases were type II C according to Grauer classification [21]. The average age of included patients was 51.8 years (range from 32 to 73 years). The details of the included 15 patients compared to the 93 non-salvage patients who successful performed the percutaneous anterior odontoid screw fixation were showed in Table 1. Pre-operative AP (anteroposterior open mouth) and lateral views and computed tomography (CT) scans were obtained to evaluate the type of the injury and upper cervical spinal anatomy of the individual patient.

**Table 1:** The details of the included patients

	Salvage patients *N* = 15	Non-salvage patients *N* = 93	*P*
Operative time (min)	53 ± 15	41 ± 19	< 0.001
Gender			
Male	12	68	0.805
Female	3	25	
Cause of injury			
Vehicle accidents	9	64	0.704
Falls	6	29	
Anderson and D’Alonzo classification			
Type II	11	72	0.985
Type III	4	21	
Grauer classification			
Type II A	0	7	< 0.001
Type II B	3	64	
Type II C	8	1	

### Ethic consideration

This study was approved by the IRB (Institutional Review Board) of the Second Affiliated Hospital and Yuying Children’s Hospital of Wenzhou Medical University, and informed consent was obtained from every participant.

#### Operative procedure

We try to perform the percutaneous anterior odontoid screw fixation on all of 108 patients: firstly, the procedure of percutaneous anterior odontoid screw fixation was described by Chi et al. [18]. Briefly, the patient was placed supine on the operative table, and the head was pulled with Gardner-Wells skull tongs to achieve anatomic reduction of the odontoid fracture. After the clear intra-operative AP (open mouth) and lateral films were obtained, an initial 10-mm skin incision was made medial to the right sternocleidomastoid muscle at the level of C4–5, blunt dissection was performed via Smith-Robinson approach to reach the anteroinferior border of C2 body using the finger or hemostat.

**Fig. 1:**
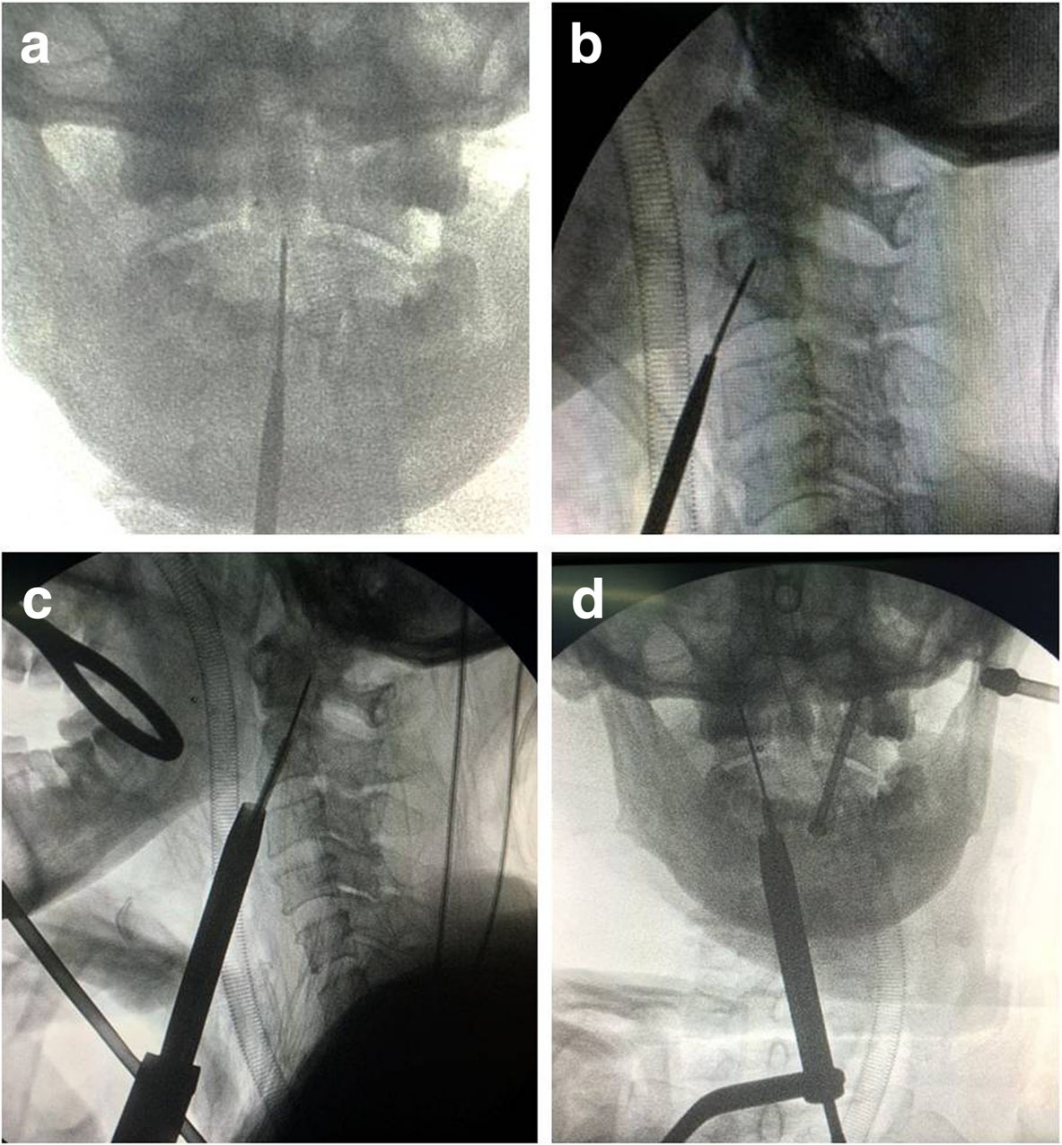
The intra-operative images. **a**, **b** Percutaneous anterior odontoid screw fixation was tried to perform first, but the trajectory of the K-wire was placed unsatisfactorily. **c**, **d** To solve the problem, percutaneous C1/2 transarticular screw fixation was performed; two 3.5-mm self-tapping cannulated transarticular screws were introduced via the previous skin wound for percutaneous anterior odontoid screw fixation

Then, the protective tube (inner diameter = 6.1 mm, outer diameter = 7.0 mm) was inserted and the guide tube (inner diameter = 1.3 mm, outer diameter = 6.0 mm) was inserted inside of the protective tube, and the guide tube was placed to the anteroinferior of C2 body; a K-wire with a 1.2-mm diameter was drilled into the odontoid process. However, in the 15 patients included in present report, the trajectory of the K-wire was placed unsatisfactorily (Fig. 1a, b); if we continue to perform percutaneous anterior odontoid screw fixation, the screw will penetrate the cortex of the odontoid process and have a higher risk of injury around the soft tissue or spinal cord or cannot provide a stiff fixation. Worse, if we try to re-drill another optimal K-wire trajectory, the initial hole will be entered inadvertently.

The percutaneous anterior odontoid screw fixation was defined failure at this time, we performed percutaneous C1/2 transarticular screw fixation on them as salvage technique. The procedure of percutaneous C1/2 transarticular screw fixation was referred to Li et al. [27]. No more skin wound was made, and only the tip of the guide tube was moved to about 5–10 mm lateral side of the mid-line anteroinferior C2 body on AP film. The angle of the K-wire was deemed to be about 20–30° to the mid-line on AP film and 20–28° to the vertical on the lateral view and drilled into C2 body, toward to the center of C1 lateral mass (Fig. 1c). Once the K-wire was placed on a satisfactory position, the guide tube will be removed, a recess for the screw head was fashioned, and a 3.5-mm self-tapping cannulated screw is then introduced over the K-wire. After the transarticular screw was well positioned, the K-wire was removed, and the same procedure was performed at the opposite site (Fig. 1d).

The patients were allowed ambulatory activities at day 2 after operation with a cervical collar, which was removed at 12 weeks after surgery.

### Pain and functional assessment

Visual Analogue Score (VAS) of neck pain and Neck Disability Index (NDI) scores were obtained at the time points of pre-operation, 3 months after operation, and final follow-up.

### Fusion assessment

CT scans were obtained at the final follow-up to assess the fusion of odontoid fractures and bony fusion bridge at the C1/2 lateral articular facet.

### Statistics

The data were collected and analyzed with the software of SPSS (version 17.0, SPSS Inc., Chicago, IL). The data of pre-operation, 3 months after operation, and final follow-up were tested by the repeated measures analysis of variance (ANOVA), The comparisons of VAS of neck pain and NDI scores between two groups were tested by independent two-sampled *t* test. The level of significance was set at *P* < 0.05.

## Results

Percutaneous C1/2 transarticular screw fixation was performed successfully in all 15 patients whose odontoid screw fixations failed. The mean operative time was 53 ± 15 min, which was 12 min more than the time cost in the 93 non-salvage patients whom were successfully performed the percutaneous anterior odontoid screw fixation (Table 1), and none of the patient had blood loss exceeding 20 ml. No technique-related complications (such as nerve injury, spinal cord injury, esophageal injury, and other around soft tissue injure) occurred. All of the 15 patients were followed up for an average of 30.5 months (range from 24 to 49 months); no screw loose or breakage was occurred, all of the odontoid fractures achieve radiographic fusion, and bony fusion bridge could be observed at the C1/2 lateral articular facet on 9/15 patients.

### Pain and functional outcomes

For all the included 15 salvage patients, VAS of neck pain was relieved significantly from 5.93 ± 1.20 at pre-operation to 1.38 ± 0.63 at 3 months after operation (*P* = 0.000) and maintained at the final follow-up, with 1.29 ± 0.55 (*P* = 0.420). Similar pain relief was achieved at the 93 non-salvage patients, with 6.29 ± 1.44 at pre-operation to 1.44 ± 1.01 at 3 months after operation (*P* = 0.000), and maintained at the final follow-up, with 1.36 ± 0.91 (*P* = 0.451). When compared between the 15 salvage patients and 93 non-salvage patients, no statistically significant difference was found at pre-operation (*P =* 0.363), 3 months after operation (*P =* 0.804), and final follow-up (*P =* 0.738) (Table 2).

**Table 2:** The pain and functional outcomes of the included 15 patients

	Salvage patients *N* = 15	Non-salvage patients *N* = 93	*P*
VAS of neck pain			
Pre-operation	5.93 ± 1.20	6.29 ± 1.44	0.363
3 months after operation	1.38 ± 0.63*	1.44 ± 1.01*	0.804
Final follow-up	1.29 ± 0.55**	1.36 ± 0.91**	0.738
NDI score			
Pre-operation	34.53 ± 4.94	35.35 ± 5.89	0.610
3 months after operation	3.73 ± 2.25***	4.20 ± 2.90***	0.550
Final follow-up	3.13 ± 2.95****	4.42 ± 2.80****	0.104

*Both *P* = 0.000 (compared to pre-operation); **P = 0.420 and *P* = 0.451 (compared to 3 months after operation), respectively; ***both *P* = 0.000 (compared to pre-operation; ****P = 0.369 and *P* = 0.494 (compared to 3 months after operation), respectively

The NDI scores was improved significantly from 34.5 ± 4.94 at pre-operation to 3.73 ± 2.25 at 3 months after operation (*P* = 0.000) and maintained at the final follow-up, with 3.13 ± 2.95 (*P* = 0.369) in the 15 salvage patients. And similarly, NDI improved from 35.35 ± 5.89 at pre-operation to 4.20 ± 2.90 at 3 months after operation (*P* = 0.000) and maintained at the final follow-up, with 4.42 ± 2.80 (*P* = 0.494) in the 93 non-salvage patients. No statistically significant difference was found at pre-operation (*P* = 0.610), 3 months after operation (*P* = 0.550*)*, and final follow-up (*P* = 0.104) (Table 2).

#### Case presentation 1

**Fig. 2:**
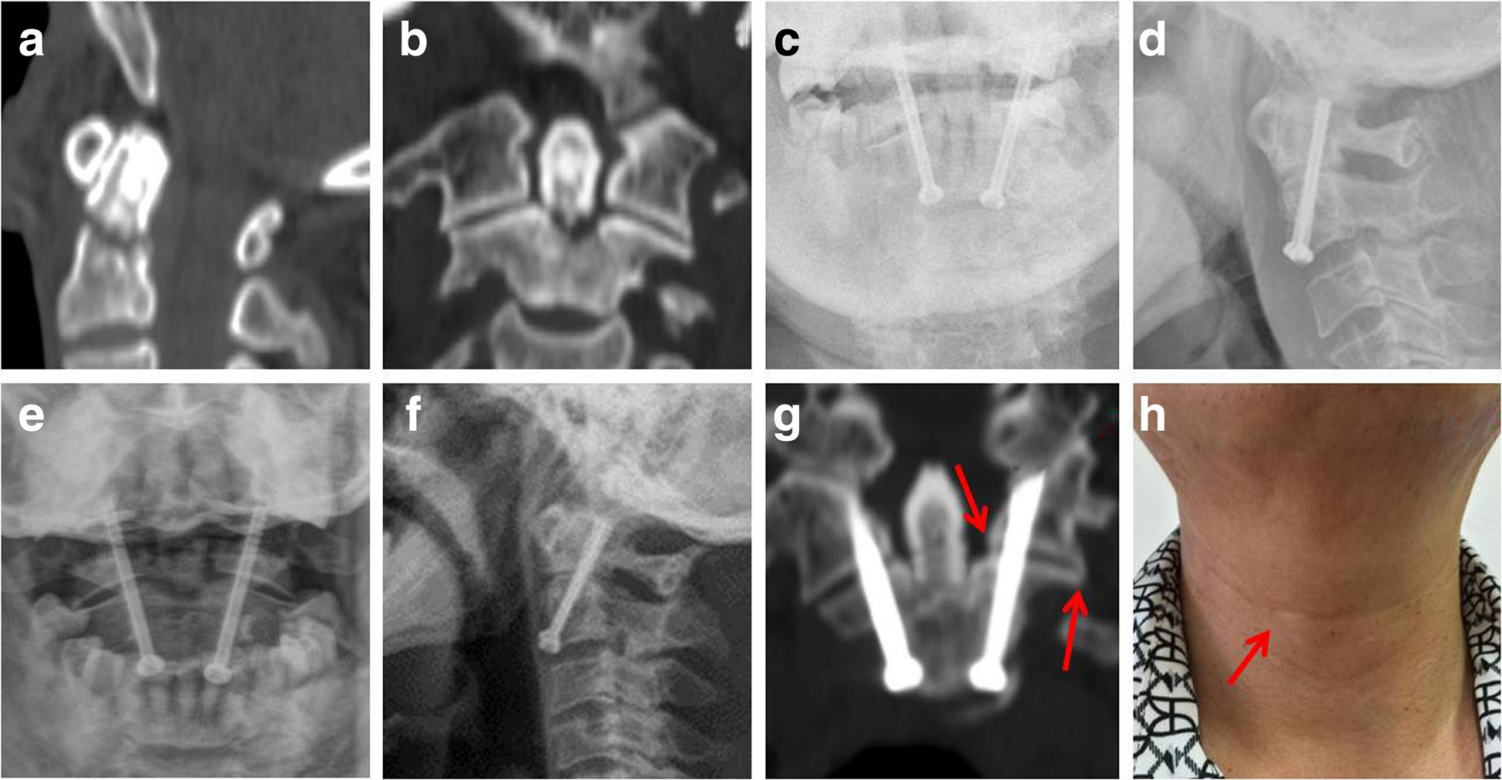
**a**, **b** The pre-operative 2D CT images of a 73-year-old man showed type II odontoid fracture. **c**, **d** Percutaneous anterior odontoid screw fixation was failed on him, and percutaneous C1/2 transarticular screw fixation was performed as salvage technique. **e**, **f** Thirty-eight months after operation, no screw loose or breakage was observed. **g**, **h** Radiographic bony fusion was observed at odontoid fracture site, and bony fusion bridge was observed at the C1/2 lateral articular facet (**g**, arrow), with only about 10-mm skin scar (**h**, arrow)

A 73-year-old man presented to the emergency department after a fall from about 2 m high place, complaining of neck pain and right limb numbness. The pre-operative 2D CT images (Fig. 2a, b) showed type II odontoid fracture, and we plan to treat him with percutaneous anterior odontoid screw fixation; however, the K-wire trajectory was unsatisfactory, and at last, percutaneous C1/2 transarticular screw fixation was performed on him (Fig. 2c, d). The neck pain was immediately relieved after operation, and right limb numbness disappeared at about 2 weeks after operation. Thirty-eight months after operation, no screw loose or breakage was observed (Fig. 2e, f), radiographic bony fusion was observed at odontoid fracture site, and bony fusion bridge was observed at the C1/2 lateral articular facet (Fig. 2g, arrow), with only about 10 mm skin scar (Fig. 2h, arrow).

#### Case presentation 2

**Fig. 3:**
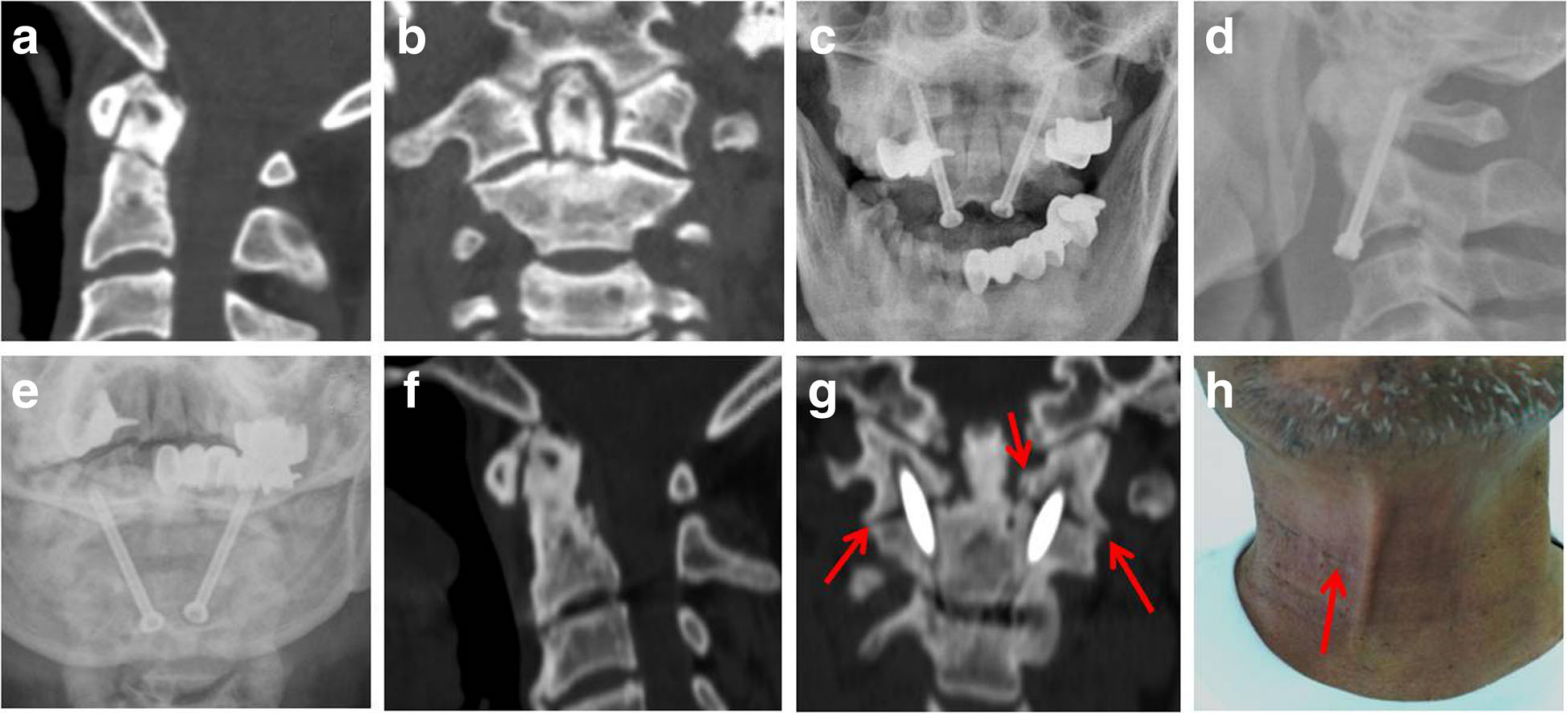
**a**, **b** The pre-operative 2D CT images of a 57-year-old man showed type II odontoid fracture. **c**, **d** Percutaneous anterior odontoid screw fixation failed, and percutaneous C1/2 transarticular screw fixation was performed on him as salvage technique. **e** Thirty-one months after operation, no screw loose or breakage was observed. **f**, **g** Bony fusion was observed at the odontoid fracture site, and bony fusion bridge was also observed at the C1/2 lateral articular facet on 2D CT images (**g**, Arrow); **h** only about 10 mm skin scar at his neck (arrow)

A 57-year-old man was sent to the emergency department after a vehicle accident and experienced neck pain and both hands’ numbness. The pre-operative 2D CT images (Fig. 3a, b) showed type II odontoid fracture. We planned to treat him with percutaneous anterior odontoid screw fixation too but failed, and percutaneous C1/2 transarticular screw fixation was performed on him as salvage technique (Fig. 3c, d). The neck pain was immediately relieved after operation, and hand numbness disappeared at about 4 weeks after operation. Thirty-one months after operation, no screw loose or breakage was observed (Fig. 2e, f), radiographic bony fusion was observed at the odontoid fracture site (Fig. 3f, g), and bony fusion bridge was also observed at the C1/2 lateral articular facet (Fig. 3f, g, arrow), with only about 10 mm skin scar too (Fig. 3h, arrow).

## Discussion

The odontoid fracture remains a challenging injury in spine injury because of the high risk of potential complications owing to the complex cranio-cervical anatomy. Many conservative treatments including halo vests and cervical orthosis were reported [28, 29]. However, the Anderson and D’Alonzo type II fractures and shallow type III odontoid fractures [3] were recognized as mechanically unstable with a high risk of nonunion and mortality [4, 30, 31], and surgical intervention was recommended [32–34].

In 1982, Bohler [14] described using the anterior odontoid screw fixation in treatment of odontoid fracture, and this technique was widely used after then and could preserve the C1/2 rotational movement, with easier patient position, less tissue trauma (because of the natural gap of the Simth-Robinson approach), and high fusion rates [23, 35–37]. Hashizume et al. [38] reported that using endoscopic system to minimize the anterior skin wound avoid soft tissue injury, Kazan et al. [39] described the technique details of percutaneous anterior odontoid screw fixation on cadaveric specimen, and Chi et al. [18] and Wang et al. [19] reported the results of clinical investigation of percutaneous anterior odontoid screw fixation for odontoid fractures, found that percutaneous technique had the advantages of being minimally invasive, minimizing blood loss, having shorter skin wound, and having quick recovery.

However, the anterior odontoid screw fixation is not suitable for all types of odontoid fractures and is not always applied successfully in all odontoid fracture patients. Salem et al. [25] reported a healthy 66-year-old male who suffers odontoid fracture after falling off a bicycle, and they performed anterior odontoid screw fixation on him first; however, the initial odontoid screw failed to maintain the interfragmentary compression achieved intra-operatively, and therefore, they did anterior C1/2 transarticular screw fixation as revision procedure on him. Ni et al. reported [26] using posterior reduction and temporary fixation as salvage maneuver to anterior screw fixation for 22 odontoid fractures patients, which they felt that the anterior odontoid screw fixation was hard or could not performed. In this study, 15 in 108 patients failed to percutaneous anterior odontoid screw fixation, we did not choose the posterior approach and only use two anterior C1/2 transarticular screws to stabilize the atlantoaxial joint.

Anterior C1/2 transarticular screw fixation was firstly reported for patients with atlantoaxial instability in 1971 by Barbour [40]. And Lu et al. [41] studied the anatomic parameters of anterior C1/2 transarticular screw fixation on 30 dried human cervical spines and proved the feasibility of anterior C1/2 transarticular screw fixation. Compared to the posterior C1/2 transarticular screw fixation (Magerl technique), anterior C1/2 transarticular screw fixation had less risk of vertebral artery injury [42]. And Elgafy et al. [43] reported that about 10–23% of patients who required atlantoaxial arthrodesis were not suitable for posterior Magerl technique because of anatomic variations of the vertebral artery; in the study of Lau et al. [44], the rate of patients with anatomic variations that are not suitable for posterior Magerl technique was approximately 40, and anterior C1/2 transarticular screw fixation was safer for them. Moreover, the human cadaveric biomechanical study showed that the anterior C1/2 transarticular screw fixation had comparable biomechanical properties to the posterior C1/2 transarticular screw fixation [45, 46].

Additionally, the 15 patients in this study were planned to be treated by anterior odontoid screw fixation, the patients were already placed supine on an operative table, and an about 10-mm anterior skin wound was already made but percutaneous anterior odontoid screw fixation fails. If we chose a posterior approach as the salvage technique, we need to change the patient’s position and make a longer posterior skin wound. And in this study, we found that the change to percutaneous anterior C1/2 transarticular screws only adds a mean 12-min operative time. There is no need to change the patients’ position and add posterior wound incision. Therefore, we suggest that salvage of percutaneous anterior odontoid screw fixation through percutaneous anterior C1/2 transarticular screw fixation had many advantages: (1) avoid changing patient’s position; (2) avoid other more skin wound; (3) the minimally invasive was similar to the percutaneous anterior odontoid screw fixation only one 10-mm skin wound; (4) good results with a high fusion rate, 15/15 (100%) odontoid fusion, some of the patients with bony fusion bridge observed at the C1/2 lateral articular facet, for some other patients without the obvious bony fusion bridge at the C1/2 lateral articular facet, no screw breakage occurred, and space of the C1/2 lateral articular facet was decreased and tend to grow the bony bridge.

Same to some other minimally invasive spinal surgical technique, the percutaneous anterior C1/2 transarticular screw fixation had its limitations. Firstly, the group size is small, because all of the 15 salvage patients are failure of anterior odontoid screw fixation; secondly, the learning curve of this technique was longer than the traditional surgical technique; thirdly, this technique had the potential risk to injure the anterior soft tissue (carotid artery, esophagus, and trachea), and our experience was that the tip of the guide tube must be pressed tightly to the front of the C2 body to avoid allowing the soft tissue to enter the space between the tip of guide tube and C2 vertebral body.

## Conclusion

Percutaneous anterior C1/2 transarticular screw fixation is a good alternative salvage technique if the percutaneous odontoid screw fixation is failure for odontoid fracture. And it is a minimally invasive, feasible, and safe technique.

## Abbreviations

C1/2: The first cervical spine/the second cervical spine
VAS: Visual Analogue Score
NDI: Neck Disability Index
ASIA: American Spinal Injury Association
CT: Computed tomography
